# PD-1 and TIGIT Are Highly Co-Expressed on CD8^+^ T Cells in AML Patient Bone Marrow

**DOI:** 10.3389/fonc.2021.686156

**Published:** 2021-08-18

**Authors:** Ling Xu, Lian Liu, Danlin Yao, Xiangbo Zeng, Yikai Zhang, Jing Lai, Jun Zhong, Xianfeng Zha, Runhui Zheng, Yuhong Lu, Minming Li, Zhenyi Jin, Sudheendra Hebbar Subramanyam, Shaohua Chen, Xin Huang, Yangqiu Li

**Affiliations:** ^1^The Clinical Medicine Postdoctoral Research Station, Department of Hematology, First Affiliated Hospital; Jinan University, Guangzhou, China; ^2^Key Laboratory for Regenerative Medicine of Ministry of Education, Institute of Hematology, School of Medicine; Jinan University, Guangzhou, China; ^3^Laboratory Center, Tianhe Nuoya Bio-Engineering Co. Ltd, Guangzhou, China; ^4^Department of Clinical Laboratory, First Affiliated Hospital, Jinan University, Guangzhou, China Guangzhou, China; ^5^Department of Hematology, First Affiliated Hospital, Guangzhou Medical University, Guangzhou, China, China; ^6^Department of Hematology, Guangdong Provincial People’s Hospital, Guangdong Academy of Medical Sciences, Guangzhou, China

**Keywords:** PD-1, TIGIT, acute myeloid leukemia, immune-checkpoints, T cell, bone marrow

## Abstract

Despite the great success of immune-checkpoint inhibitor (ICI) treatment for multiple cancers, evidence for the clinical use of ICIs in acute myeloid leukemia (AML) remains inadequate. Further exploration of the causes of immune evasion in the bone marrow (BM) environment, the primary leukemia site, and peripheral blood (PB) and understanding how T cells are affected by AML induction chemotherapy or the influence of age may help to select patients who may benefit from ICI treatment. In this study, we comprehensively compared the distribution of PD-1 and TIGIT, two of the most well-studied IC proteins, in PB and BM T cells from AML patients at the stages of initial diagnosis, complete remission (CR), and relapse-refractory (R/R) disease after chemotherapy. Our results show that PD-1 was generally expressed higher in PB and BM T cells from *de novo* (DN) and R/R patients, while it was partially recovered in CR patients. The expression of TIGIT was increased in the BM of CD8^+^ T cells from DN and R/R patients, but it did not recover with CR. In addition, according to age correlation analysis, we found that elderly AML patients possess an even higher percentage of PD-1 and TIGIT single-positive CD8^+^ T cells in PB and BM, which indicate greater impairment of T cell function in elderly patients. In addition, we found that both DN and R/R patients accumulate a higher frequency of PD-1^+^ and TIGIT^+^ CD8^+^ T cells in BM than in corresponding PB, indicating that a more immunosuppressive microenvironment in leukemia BM may promote disease progression. Collectively, our study may help guide the combined use of anti-PD-1 and anti-TIGIT antibodies for treating elderly AML patients and pave the way for the exploration of strategies for reviving the immunosuppressive BM microenvironment to improve the survival of AML patients.

## Introduction

In the last few decades, immunotherapy has emerged as the fourth pillar following surgery, radiation/chemotherapy and targeted therapy for solid tumor and leukemia therapy. One of the most effective immunotherapies includes resolving T cell dysfunction with immune checkpoint (IC) inhibitors (ICIs), such as programmed death receptor-1 (PD-1) and its ligand (PD-L1), cytotoxic T lymphocyte-associated antigen-4 (CTLA-4), and T cell immunoglobulin mucin-3 (Tim-3) (1–5). IC proteins are co-inhibitory receptors that distributed on the surface of several immune cells. After ligand binding, these regulators can transducing inhibitory signals to avoid immune injury under physiological conditions. However, under pathological conditions, such as chronic inflamation and cancer, continuous antigen stimulation and several immunosupressive factors could drive T cells to develop into a “unfitness” state called T cell exhaustion. One of the features of T cell exhaustion is the occurrence of elevated expession of several IC proteins. When these proteins bind their ligands, they reduce the anti-virus or anti-tumor effects of T cells, ultimately resulting in the immune escape of pathogens or tumors (3, 6–8).

Acute myeloid leukemia (AML) is a genetically, epigenetically, and clinically heterogeneous disease characterized by a failure in normal hematopoiesis and the accumulation of immature myeloid cells in the bone marrow (BM) and peripheral blood (PB) (9). For all AML patients (except for those with acute promyelocytic leukemia) who are medically able to tolerate chemotherapy, treatment with a 7-day infusion of cytarabine and a 3-day infusion of daunorubicin (“7^+^3”), has not changed in the past 50 years. Additionally, post-remission therapy aimed to prevent relapse of the disease. Additional cytotoxic chemotherapies (such as high or intermediate dose cytarabine), with or without targeted therapies, and allogenic hematopoietic stem cell transplantation (Allo-SCT) are the two commonly employed strategies. Several targeted therapies have been developed and approved for AML patients with special molecular and cytogenetic alterations, including venetoclax to target B-cell lymphoma 2, midostaurin, and gilteritinib to target FLT3, and ivosidenib and enasidenib to target mutant isocitrate dehydrogenase 1 and 2 (IDH1 and IDH2). The outcomes of AML patients who have a favorable or intermediate risk prognostic classification have improved with the addition of various targeted drugs; however, longer-term overall survival (OS) beyond 3–5 years remains low for adverse risk patients, particularly older AML patients (9–14). In recent studies, ICIs have demonstrated encouraging results when combined with hypomethylating agents (HMAs) in relapsed/refractory AML patients, particularly for those who were not exposed to HMA treatment, but most of the other clinical trials did not achieve a satisfactory response when using ICIs alone (15–19). One of the reasons for this discrepancy may be that the patients involved in those trials often experienced failure with multiple lines of conventional therapy, which could have a long-term impact on immunological fitness and clinical responses (20, 21). Therefore, comparing the immune status of T cells in AML patients before and after chemotherapy may help determine which type of patient could benefit from novel immune therapies. Additionally, cancer cells can orchestrate surrounding cells, such as regulatory T cells (Tregs), myeloid-derived suppressor cells (MDSCs), and plasmacytoid dendritic cells (pDCs), to construct an immunosuppressive tumor microenvironment (TME), enabling immune evasion and immunotherapy resistance (22, 23). In the BM, which is the nest of the leukemia progenitors generated and output in AML, the environment is quite complicated and resembles that of the TME in solid tumors to some degree. For example, the BM of AML patients also accumulates Treg, MDSC, and pDC cells that could inhibit the anti-leukemia immune response of T cells (24–27). Increasing evidence has shown that the BM immune environment of AML patients is profoundly altered, contributing to the severity of the disease; however, there have been limited studies comparing differences in T cell dysfunction between PB and BM. Thus, further elucidating the characteristics of the immune microenvironment in the BM of AML patients may help guide the use of immunotherapy drugs and facilitate the exploration of new immune targets.

PD-1, CTLA-4, and Tim-3 are the most well-studied IC proteins, and multiple other IC proteins are also targeted by immune checkpoint blockades or agonists in clinical research, such as lymphocyte activation Gene-3 (LAG-3), T cell immunoreceptor with immunoglobulin, and ITIM domain (TIGIT), and tumor necrosis factor receptor (OX40) (28–31). TIGIT, which is express on activated T cells, Treg cells, and NK cells, has been identified as a promising new target for cancer immunotherapy in recent years (2, 3, 7, 28, 32–34). Previously, high expression of PD-1 and TIGIT on PB T cells from AML patients has been reported (33, 35, 36), and higher PD-1 expression also detected on BM T cells in our previous study (37); however, how the expression of TIGIT and its co-expression with PD-1 changes in the BM of *de novo* (DN) AML patients remains unknown. Thus, we comprehensively compared the single and dual expression of PD-1 and TIGIT in both PB and BM T cells from AML patients at initial diagnosis. In addition, since ICI treatment typically used for patients with relapsed-refractory (R/R) disease, it is necessary to check the fitness of T cells from patients who have received chemotherapy. Thus, we also analyzed the expression of PD-1 and TIGIT in the PB and BM of AML patients at completed remission (CR) and disease relapse after chemotherapy.

## Materials and Methods

### Sample Collection and Preparation

A total of 38 PB samples and 32 BM samples from *de novo* AML patients [(median age, range), (PB: 54.5, 11-88; BM: 57.5, 14-80)] were collected, including 26 paired samples. A total of 17 PB samples and 21 BM samples from AML patients who achieved CR (PB: 46, 13-80; BM: 47, 13-73) were collected, including 16 paired samples. A total of 10 PB samples and 8 BM samples from AML patients with relapsed-refractory disease (PB: 51, 23-80; BM: 49, 23-67) were collected, including 6 paired samples. All of the patients included in our CR and R/R cohorts are transplant-naïve patients. In addition, 36 PB samples from healthy donors (53, 13-65), and 14 unpaired BM samples from hematopoietic stem cell transplantation donors or iron deficiency anemia patients (47, 17-68) underwent bone marrow aspiration served as the control group. Sample details shown in **Table 1**. All samples collected with informed consent. Ethical approval for the study was obtained from the Ethics Committee of the First Affiliated Hospital of Jinan University. All procedures were conducted according to the guidelines of the Medical Ethics Committees of the Health Bureau of the Guangdong Province in China. All samples were freshly obtained and subjected to immediate preparation.

**Table 1:** Clinical characteristics of HIs and AML patients.

Variables	HIs		DN AML		CR AML		R/R AML	
	PB	BM	PB	BM	PB	BM	PB	BM
Number	36	14	38	32	17	21	10	8
Age (years)	53	47	54.5	57.5	46	47	50	49
median, range	(13-84)	(17-68)	(11-88)	(14-80)	(13-80)	(13-73)	(23-80)	(23-67)
Gender, M/F	19/17	6/8	17/21	18/14	10/7	10/11	5/5	5/3
WBC (×10^9^/L), mean ± SD	/	/	54.92 ± 135.16	/	4.60 ± 1.77	/	9.29 ± 16.37	/
PLT (×10^9^/L), mean ± SD	/	/	81.95 ± 143.85	/	193.69 ± 133.46	/	71.00 ± 58.68	/
Blasts (%) mean ± SD,	/	/	/	63.15 ± 20.03	/	1.64 ± 2.09	/	25.59 ± 27.32
Paired sample (n)	2		27		16		6	
Subtype(n, %)								
MDS-T			3 (7.9%)	2 (6.3%)	1 (5.9%)	1 (4.8%)	2 (20%)	3 (37.5%)
M1			1 (2.6%)	0 (0.0%)	0 (0.0%)	0 (0.0%)	0 (0.0%)	0 (0.0%)
M2			13 (34.2%)	11 (34.4%)	5 (29.4%)	8 (38.1%)	1 (10%)	0 (0.0%)
M3			2 (5.3%)	4 (12.5%)	4 (23.5%)	4 (19.0%)	0 (0.0%)	0 (0.0%)
M4			6 (15.8%)	5 (15.6%)	1 (5.9%)	1 (4.8%)	3 (30%)	3 (37.5%)
M5			11 (28.9%)	9 (28.1%)	5 (29.4%)	4 (23.8%)	2 (20%)	3 (37.5%)
Unclassified			2 (5.3%)	1 (3.1%)	1 (5.9%)	2 (9.52%)	2 (20%)	3 (37.5%)

M/F, male/female; WBC, white blood cell; PLT, platelets; n, number; MDS-T, myelodysplastic syndrome transformed.

### Flow Cytometry Analysis

Cell surface staining for flow cytometry was performed using the following antibodies: CD45-BUV395 (clone HI30, BD), CD3-AF700 (clone UCHT1, BD), CD4-APC-H7 (clone RPA-T4, BD), CD8-APC-H7 (clone SK1, BD), TIGIT-PE/Dazzle™ 594 (clone A15153G, Biolegend), TIGIT-BV421 (clone A15153G, Biolegend), and PD1-BV421 (clone EH12.2H7, Biolegend). These antibodies were used to analyze surface receptors in two different panels. Isotype-matched antibodies, labeled with the proper fluorochromes, were used as negative controls. Extracellular staining was performed according to the manufacturer’s instructions. Briefly, an antibody cocktail was added to whole blood or BM samples, which were then incubated at room temperature for 15 minutes in the dark, followed by red cell lysis with lysis buffer (BD; Cat: 555899). The lysed cells were washed twice with phosphate buffer saline (PBS), and then 350 μl stain buffer was added for further flow cytometer analysis. Twenty microliters of absolute count microsphere (Thermo; Cat: C36950) were added to the samples for absolute number analysis. Cells were analyzed using a BD Fortessa flow cytometer (BD Biosciences), and data analysis performed with Flowjo 10.6 software.

### Statistical Analysis

Data were analyzed by correlation, linear regression, independent t-test, or paired t-test depending on the experimental design. Calculations were performed using GraphPad Prism, version 8.02 software. Significance is indicated as *p < 0.05, **p < 0.01, ***p < 0.001, ****p < 0.0001. Values of *p* < 0.05 were considered significant.

## Results

### The Distribution of PD-1 and TIGIT in the PB and BM of AML Patients During Diagnosis, Complete Remission, and in Relapsed-Refractory Patients

**Figure 1:**
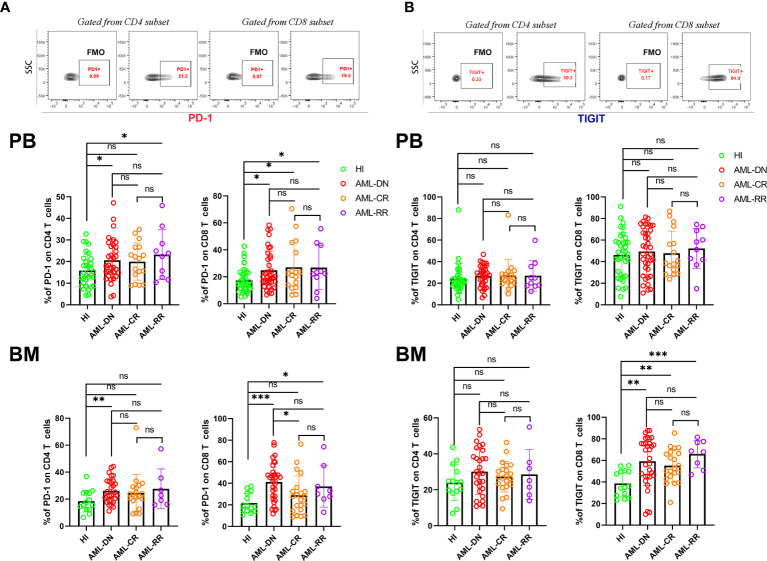
T cells from the PB and BM of DN AML and R/R AML patients generally have high expression of PD-1, and BM CD8^+^ T cells from DN, CR, and R/R patients have increased TIGIT expression. **(A)** The top figure shows the gating strategy for PD-1 in the CD4^+^ and CD8^+^ populations by flow cytometry. The frequency of PD-1 by subset in the PB of HIs (CD4, n=33; CD8, n=36) and patients in the AML-DN (CD4, n=34; CD8, n=38), AML-CR (n=17), and AML-R/R (n=10) cohorts (*Upper*); The frequency of PD-1 by subset in the BM of HIs (n=14), and patients in the AML-DN (CD4, n=29; CD8, n=32), AML-CR (n=21), and AML-R/R (CD4, n=7; CD8, n=8) cohorts (*Lower*). The PD-1 expression frequency generally increased in T cells from DN and R/R patients. **(B)** The top figure shows the gating strategy for TIGIT in the CD4^+^ and CD8^+^ populations by flow cytometry. The frequency of TIGIT by subset in the PB (*Upper*) and BM of HIs, and patients in the AML-DN, AML-CR, and AML-R/R cohorts (*Lower*); The TIGIT expression frequency increased in BM CD8^+^ T cells from DN, CR, and R/R patients compared with HIs. The *p* values shown are from independent t-tests between groups. *p < 0.05, **p < 0.01, ***p < 0.001, ns denotes not significant.

We first compared the PD-1 and TIGIT expression frequency on CptD4^+^ and CD8^+^ T cells from the PB and BM of AML patients in the DN, CR, and R/R stages. The results demonstrated that PD-1 expression increased on both PB CD4^+^ (20.63% *vs* 15.86%, p=0.029) and CD8^+^ (24.77% *vs* 17.49%, p=0.011) T cells in DN patients when compared with healthy individuls (HIs), while the same result was found when comparing the BM CD4^+^ (26.17% *vs* 18.48%, p=0.010) and CD8^+^ (41.17% *vs* 21.68%, p=0.0003) T cell populations. When patients achieved CR, we found that PD-1 expression recovered in most T cell populations except for the PB CD8^+^ population (27.00% *vs* 17.49%, p=0.014). In addition, we also detected higher PD-1 expression on PB CD4^+^ (23.22% *vs* 15.86%, p=0.023) and CD8^+^ (26.84% *vs* 17.49%, p=0.021) T cells as well as BM CD8^+^ (37.14% *vs* 21.68%, p=0.018) T cells from R/R patients when compared with HIs (**Figure 1A**). Unlike the high expression of PD-1 on both PB and BM T cells in DN AML patients, TIGIT expression was increased only in the BM CD8^+^ (59.18% *vs* 38.69%, p=0.003) T cell population. Interestingly, the expression of TIGIT was also higher in the BM CD8^+^ population from CR (55.08% *vs* 38.69%, p=0.002) and R/R (65.94% *vs* 38.69%, p<0.0001) patients compared with HIs (**Figure 1B**). The above results indicated that higher expression of PD-1 in PB and BM T cells is a characteristic of patients with DN AML and might be a sign of disease relapse, while PD-1 expression recovery might be a favorable signal of disease remisson. Whether PD-1 expression could predict the prognosis or survival of AML patients needs to be confirmed in a future study.

### Age Correlation of PD-1 and TIGIT Expression on PB and BM T Cells

**Figure 2:**
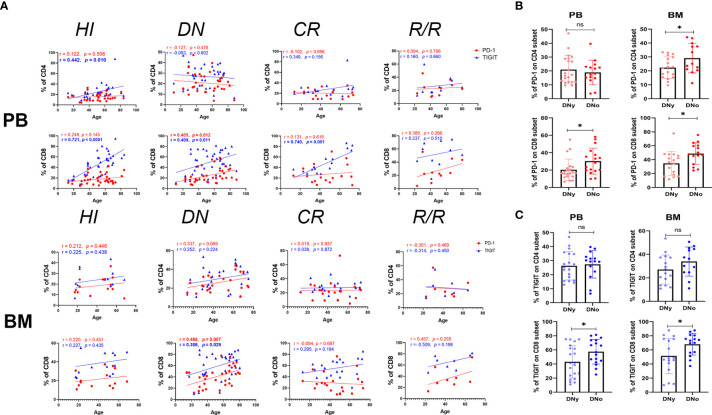
PD-1^+^ and TIGIT^+^ CD8^+^ T cells increase in the BM of older DN-AML patients. **(A)** The upper panel shows the correlation between the frequency of PD-1 (red dots) or TIGIT (blue triangles) and age in the PB CD4^+^ and CD8^+^ T cell populations in HIs (CD4, n=33; CD8, n=36) and patients in the AML-DN (CD4, n=34; CD8, n=38), AML-CR (n=17), and AML-R/R (n=10) cohorts; the lower panel shows the correlation between the frequency of PD-1 (the red dots) or TIGIT (blue triangles) and age in the BM CD4^+^ and CD8^+^ T cell populations in HIs (n=14) and patients in the AML-DN (CD4, n=29; CD8, n=32), AML-CR (n=21), and AML-R/R (CD4, n=7; CD8, n=8) cohorts. Spearman’s nonparametric test used to test for correlations between each group. **(B)** Comparison of the expression of PD-1 on CD4^+^ and CD8^+^ T cells from the PB of DNy (CD4, n=19; CD8, n=21) and DNo (CD4, n=15; CD8, n=17) (younger and older than 60 years) patients (Left) from the BM of DNy (CD4, n=16; CD8, n=17) and DNo (CD4, n=13; CD8, n=15) patients (Right). **(C)** Comparison of the expression of TIGIT on CD4^+^ and CD8^+^ T cell subsets from the PB and BM of DNy and DNo AML patients. The sample cases used for TIGIT are the same as those used for the PD-1 comparison. The *p* values shown in B and C are from the independent student’s t-test. *p < 0.05, ns denotes not significant.

TIGIT was previously reported to have increased expression in blood T cells of elderly healthy individuals (38); thus, we analyzed the correlation between age and the expression frequency of PD-1 and TIGIT on PB and BM CD4^+^ and CD8^+^ T cells in each group. We found that PD-1 had no age correlation with any T cell subset in the HI group; however, in the DN group, the expression of PD-1 on PB and BM CD8^+^ T cells was positively correlated with age. In addition, the expression of PD-1 on BM CD4^+^ T cells also demonstrated a trend toward a positive correlation (**Figure 2A**). These results suggest that the expression of PD-1 in elderly AML patients may be even higher than that in young patients, which may weaken the anti-leukemia T cell response of elderly patients. Elderly AML generally defined as AML in a patient who is greater than 60 years of age. Hence, we further compared the expression of PD-1 on CD4^+^ and CD8^+^ T cells in the PB and BM of young (< 60 years) and elderly (>= 60 years) AML patients. The results demonstrated that PD-1 is expressed higher on all T cell subsets except for the CD4^+^ subset in the PB of elderly AML patients (**Figure 2B**). As shown in **Figure 2A**, there was also no correlation between age and PD-1 expression for any of the T cell subsets in the CR and R/R groups.

Regarding the expression of TIGIT, we found the exact age correlation for its expression on CD4^+^ and CD8^+^ T cells in HI PB, which was dramatically evident in the CD8^+^ population. Additionally, we found that the positive correlation between TIGIT and age in the PB CD4^+^ T cell population lost in the DN, CR, and R/R groups. In contrast, in the PB CD8^+^ T cell population, it was diminished in the DN group but recovered in the CR group. Considering the strong age correlation of TIGIT in the PB of HIs, we further dissected the HI, DN, and CR groups into young and elderly cohorts and compared the expression of TIGIT on each subset. The results demonstrated that in the cohorts under age 60, the frequency of TIGIT on the CD4^+^ T cells of DN AML patients was higher than that on HIs (26.31% *vs* 20.14%, p=0.038); however, there was no difference in other comparisons between AML and HIs in the same age cohorts (**Supplementary Figure 1**). Regarding the expression of TIGIT on HI BM T cells, there was no age correlation found in either the CD4^+^ or CD8^+^ population, but a significant age correlation was found for the DN AML group (**Figure 2A**). Further analysis revealed that the frequency of TIGIT in the CD8^+^ population in BM was higher in elderly *vs*. younger DN AML patients (**Figure 2C**).

In summary, we found that the frequency of PD-1 expression on T cells in HIs had no age correlation in PB or BM; however, strikingly, there was higher PD-1 expression in elderly AML patients. Moreover, the above results confirmed that the expression of TIGIT on T cells is closely correlated with age in PB but not BM. However, higher TIGIT expression is still detected in the CD4^+^ population of DN patients under 60 years. In addition, relatively higher TIGIT expression was also detected in the BM CD8^+^ T cell subset of elderly DN patients.

### Higher PD-1 and TIGIT Expression Detected in the BM of DN and R/R AML Patients

**Figure 3:**
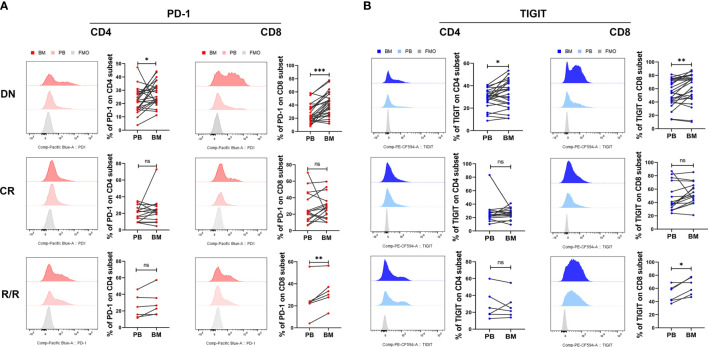
BM CD4^+^ and CD8^+^ T cells from DN-AML and R/R-AML patients have a higher percentage of PD-1- and TIGIT-expressing T cells than matched PB. **(A)** The flow-cytometry analysis detected an increase in the frequency of PD-1-expressing CD4^+^ and CD8^+^ T cells in the BM of DN-AML patients compared with matched PB (CD4, n=24; CD8, n=27); the expression of PD-1 on CD4^+^ and CD8^+^ T cells between PB and BM was not different in the CR-AML cohort (CD4, n=15; CD8, n=16). Increased PD-1 expression was also detected in BM CD8^+^ T cells from R/R patients (n=6). **(B)** Flow-cytometry analysis detected an increased frequency of TIGIT-expressing CD4^+^ and CD8^+^ T cells in the BM of DN-AML patients compared with matched PB (CD4, n=24; CD8, n=27); the expression of TIGIT on CD4^+^ and CD8^+^ T cells between PB and BM was not different in the CR-AML cohort (CD4, n=15; CD8, n=16); increased TIGIT expression was also detected in the BM CD8^+^ T cells of R/R patients (n=6). The *p* values in **(A, B)** were calculated by the paired student’s t-test. *p < 0.05, **p < 0.01, ***p < 0.001, ns denotes not significant.

Previous studies have found that the leukemia BM micro- environment possesses several immune suppressive factors that may protect malignant hematopoietic stem cells from immunological surveillance. We and others have also reported that PD-1 increased in BM CD8^+^ T cells from DN AML patients, which may promote immune evasion for leukemia cells in the BM (36, 37, 39, 40). In this study, we further compared the expression of PD-1 and TIGIT in paired PB and BM from DN, CR, and R/R patients. The results demonstrated that both the CD4^+^ and CD8^+^ T cell subsets in the BM of DN AML patients possessed a higher percentage of PD-1 (**Figure 3A**) and TIGIT (**Figure 3B**) when compared with corresponding PB, and the increase in the CD8^+^ population was most apparent. In addition, the above differences did not exist for CR patients and remained in the CD8^+^ population for R/R patients. These results indicate that the BM microenviroment of AML patients possesses an increased number of immunosupressive factors compared with PB, and reviving the immunosupressive microenviroment might be a key factor for treating AML and preventing relapse.

### Higher Co-Expression of PD-1 and TIGIT was Detected in the DN and R/R AML Cohorts, and DN-AML Patients Accumulated More PD-1^+^TIGIT^+^ CD8^+^ T Cells in BM Than PB

**Figure 4:**
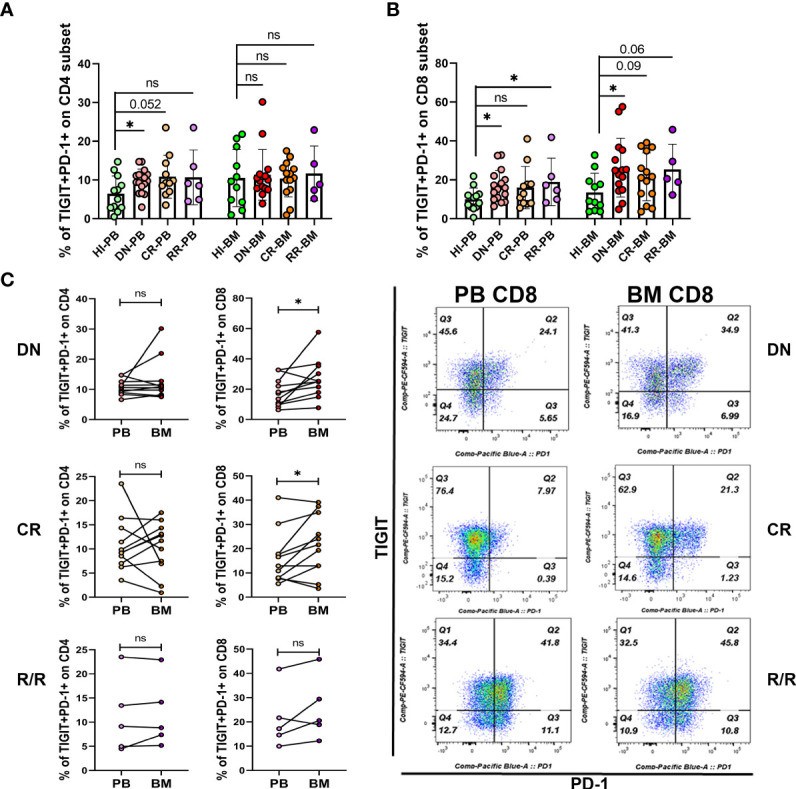
Co-expression of PD-1 and TIGIT is increased in the PB and BM T cells of DN-AML patients. **(A)** Shown is the co-expression of PD-1 and TIGIT in PB and BM CD4^+^ T cells from HIs (PB, n=12; BM, n=11) and patients in the DN-AML (PB, n=16; BM, n=15), CR-AML (PB, n=11; BM, n=14), and R/R-AML (n=5) cohorts. **(B)** Co-expression of PD-1 and TIGIT in PB and BM CD8^+^ T cells from HIs and patients in the DN-AML, CR-AML, and R/R-AML cohorts. The cases in the figure are the same as those in the CD4^+^ population. The *p* values in **(A, B)** were calculated by the independent student’s t-test. **(C)** Comparison of PD-1 and TIGIT co-expression between the paired PB and BM CD4^+^ and CD8^+^ subsets. Higher co-expression existed in the BM CD8^+^ T cell from most of the DN-AML and CR-AML patients. The *p* values shown in C were calculated by the paired student’s t-test. *p < 0.05, ns denotes not significant.

Co-expression of several IC proteins is a characteristic of exhausted T cells. A high percentage of PD-1^+^TIGIT^+^CD8^+^ T cells was found in the PB of DN AML patients with the exhaustion phenotype; however, it remains unknown whether they change in the BM and in patients who achieve CR or those with R/R disease. Thus, we further examined the co-expression of PD-1 and TIGIT in the PB and BM of each cohort. As shown in **Figure 4A**, higher co-expression of PD-1 and TIGIT only detected on PB CD4+ T cells from DN patients, while, as shown in **Figure 4B**, higher co-expression detected on PB CD8^+^ T cells from DN and R/R AML patients but not on the cells in the CR group. However, increased co-expression was also found in the BM CD8^+^ T cell population of AML patients in all disease conditions. However, statistical significance was found only for DN patients based on the limited CR and R/R patient samples analyzed (**Figure 4B**). We further compared the co-expression of PD-1 and TIGIT between PB and BM in AML patients at different disease stages. We found no significant co-expression difference in the CD4^+^ population in any of the AML sub-groups (**Figure 4C**, left). In terms of the CD8^+^ population, higher co-expression was detected in the DN and CR cohorts (**Figure 4C**, right). These results further suggest a more immunosupressive BM microenvironment in AML patients at initial diagnosis and relapse.

## Discussion

Previous studies have reported the expression of PD-1 and TIGIT in AML patients at diagnosis and after hematopoietic stem cell transplantation (33, 35–37, 41, 42); however, most of these studies were only concerned about T cells in the PB. Some of these studies ignored the impact of patient age; thus, controversial results exist. In this study, we comprehensively compared the distribution of PD-1 and TIGIT, two of the most well-studied IC proteins in PB and BM T cells from AML patients at different disease stages. The results demonstrated that PD-1 is highly expressed in almost all CD4^+^ and CD8^+^ T cells in the PB and BM of DN AML patients and demonstrates higher expression in the elderly than younger patients. Expression in the BM was higher than that in PB. When patients achieve CR after induction chemotherapy, most of T cells can restore the average expression level of PD-1 except for the CD8^+^ T cells in PB. In addition, patients who were not response to or relapsed from chemotherapy maintained a high expression of PD-1 in most of the T cell subsets except for CD4^+^ T cells in BM. These results indicated that the function of T cells in elderly patients at first diagnosis might be worse than that in younger patients, which may be a reason for their chemotherapy intolerance, but this may be an advantage for receiving ICI therapy. Although most of the clinical trials using PD-1/PD-L1 inhibitors have been administered to R/R AML patients who experienced intensive induction chemotherapy at least twice (16, 32, 43, 44), a multi-center phase 2 study (NCT02845297) of pembrolizumab (PD-1–blocking antibody) and azacitidine (AZA) in patients with R/R AML and a small cohort of newly diagnosed AML patients (median age and range: 75, 67-83 years) suggested a favorable overall response rate (ORR; 71%) and survival for newly diagnosed and unfit older AML patients.

Moreover, this study also found that the AZA/pembro combination is safe, feasible, and well tolerated by these elderly patients (45). Moreover, a higher survival rate when using drugs targeting PD-1 in metastatic melanoma patients between the ages of 70 and 80 was reported in a large population-based Danish patient cohort (1,082 cases) (46). In addition to age, cytogenetic abnormalities are another critical prognostic factor for AML. Thus, analyzing changes in T cell status in patients with different karyotypes and gene mutations is also essential. Previously Williams et al. reported that PD-L1 is higher expressed in BM blasts from patients with *TP53* mutation and also have a higher expression trend in patients with adverse cytogenetics; however, they did not find any correlation between cytogenetic abnormalities and the expression of IC proteins in T cells (36). We also analyzed the expression of PD-1 and TIGIT in patients with or without *FLT3-ITD* mutation and complex karyotype. However, there was no difference to be found in either group (data not shown). To explore the relationship of PD1 and TIGIT expression on T cells with other gene mutations, such as *TP-53* and IDH1/2, would require a larger cohort study in the future.

The high expression of PD-1/PD-L1 is an essential indicator for effective ICI therapy; however, whether sufficient tumor-infiltrating T cells in the tumor microenvironment is also a prerequisite (32). We also analyzed the absolute numbers of T cells in the PB and BM samples included in this study. We found increased numbers of infiltrating T cells in the BM of DN patients, and CR and R/R patients had a lower T cell count in the PB and comparable numbers of T cells in the BM compared with HIs (**Supplementary Figure 2**). These results suggest that the T cells were activated and responded to leukemia antigen stimulation in DN patients. However, most of them may be functionally impaired by expressing IC proteins and other immunosuppressive factors. When receiving induction chemotherapy, most of the T cells were killed simultaneously by the unspecific cytotoxicity of the chemotherapy drugs; thus, there might not be sufficient numbers of T cells to respond to ICI treatment. This reason may be one of the causes for anti-PD-1 treatment without satisfactory effects in R/R patients; however, whether a single anti-PD-1 agent would affect the overall survival (OS) of untreated younger AML patients remains unknown. Recently, a phase 2 study (NCT02464657) using nivolumab combined with induction cytarabine plus idarubicin produced an ORR of 80% and a median OS of 18.54 months in untreated, younger AML patients. Interestingly, responding patients who continued idarubicin, cytarabine, and nivolumab beyond remission had similar overall survival and event-free survival compared with those bridged to allogeneic stem cell transplantation, suggesting the possibility of nivolumab efficacy in restoring host antitumor immune surveillance. Moreover, the authors also found that a higher prevalence of CD3-positive T cells in the bone marrow before the therapy administration appeared to predict the response (47). This study, combined with our findings here, may shed light on using anti-PD-1 as a single agent or in combination with other drugs for treating DN AML patients, particularly elderly DN AML patients. Previously, two studies also compared the absolute number of T cells in AML patients; one used flow-cytometry and found a higher CD3^+^ T cell number in the PB of DN AML patients compared with age-matched HIs. The different results reported here may be because we used anti-CD45 to exclude the impact of many leukemia cells in the samples (48). In another study, the authors used immunochemistry to compare the CD3^+^ T cell number in the BM and found a comparable amount of CD3^+^ T cells between DN patients and HIs (36). Considering the high sensitivity of flow cytometry in minimal residual disease detection and the greater number of samples analyzed in our study (31 *vs* 14), we believe that our results have higher accuracy. Whether the increased absolute count of T cells in the PB and BM of DN AML patients would predict the outcome and survival of DN AML patients is worth further exploration with a large cohort.

With regards to the expression of TIGIT, we confirmed Yangzi Song’s result that the TIGIT^+^ cell frequencies among PB CD8^+^ T cells were significant age correlated, whereas CD4^+^ T cells exhibited exhibited a weak correlation (38). Based on this age-induced change in PB T cells, the different expression of TIGIT in PB T cells between AMLy and AMLo patients is most likely to be a distinction that also exists in the HIy and HIo groups. In 2016, Yaxian Kong had reported that TIGIT expression elevated in the PB CD8^+^ T cells of AML patients, and high TIGIT was associated with poor clinical outcome in AML (49); however, we only found higher expression of TIGIT on PB CD4^+^ T cells of DN AML patients compared with HIs in the younger age group and on PB CD8^+^ T cells in both young and older patients. This inconsistency may arise from the average age of their healthy cohort appears to be younger than that of the AML cohort (51 *vs.* 60). Interestingly, the expression of TIGIT on BM T cells demonstrated no age correlation for HIs. Thus, we speculate that TIGIT function in BM T cells might be different from that in PB. Thus, the elevated TIGIT expression on the BM CD8^+^ T cells of DN, CR, and R/R patients may be mainly impacted by the leukemia microenvironment itself, while the BM microenvironment of elderly patients may further exacerbate the high expression of TIGIT. Although the immunosupressive functions of TIGIT have been demonstrated many times, whether its high expression on the BM CD8^+^ T cells of AML patients correlates with prognosis and survival remains to be seen in the future. In addition, further studies should elucidate the functional heterogeneity of TIGIT^+^ T cells co-expressed with other IC proteins and with its competitive activation receptor CD226, considering the complex mechanisms of TIGIT in suppressing T cell function.

Co-expression of several IC proteins is usually thought to be one of the characteristics of T cell exhaustion. Two previous studies have reported higher dual expression of PD-1 and TIGIT in PB T cells from DN AML patients, and functional experiments supported that PD-1^+^TIGIT^+^ CD8^+^ T cells were functionally exhausted (35, 50). In this study, we further found that the percentage of PD-1 and TIGIT double-positive T cells not only increased in PB but also in BM CD8^+^ T cells from DN AML patients. In addition, it was shown that there was an increase in PB and a trend toward an increase in BM CD8^+^ T cells in R/R AML patients. All these results indicated that a more immunosuppressive microenvironment might exist in the BM of AML patients. Previously Jia’s study found that intracellular expression of Granzyme B in BM CD8^+^ T cells was significantly lower compared with that of PB, which supports our results (51); however, a study from Lamble et al. reported that there was no significant proliferation difference between BM and PB CD8^+^ T cells from AML patients (52), which may be due to the high patient-to-patient variability when looking into their data. Such individual differences also existed in our study; thus, to precisely understand the dysfunctional status of BM T cells in each AML patient, a comprehensive assessment of the immune checkpoints expression, cytokine secretion, and proliferation capacity of paired PB and BM samples is needed. In addition, whether these results support the combined use of anti-PD-1 and anti-TIGIT ICIs for treating DN and R/R patients remains to be seen.

Taken together, in addition to the previously reported high expression of PD-1 and co-expression of PD-1 and TIGIT in the PB of DN AML patients and increased PD-1 expression in the BM of DN AML patients, we further identified the accumulation of TIGIT^+^ CD8^+^ T cells in the BM of DN, CR, and R/R patients. Moreover, we found a higher percentage of PD-1 and TIGIT co-expressing CD8^+^ T cells in the BM of DN AML patients. These results support the idea that the BM microenvironment of AML patients possesses many more immunosupressive factors than PB. Future treatment strategies focused on reviving the immunosuppressive BM microenvironment may improve AML patients’ survival. Moreover, we also found that increased PD-1 and TIGIT single-positive T cells exist in the PB and BM of older AML patients compared with younger patients, suggesting that T cell function in elderly patients might be even worse than that in younger patients. This finding may partially explain the chemotherapy intolerance of older AML patients, which brings hope for using ICIs to treat DN elderly AML patients who may have sufficient T cells to respond.

## Data Availability Statement

The raw data supporting the conclusions of this article will be made available by the authors, without undue reservation.

## Ethics Statement

The studies involving human participants were reviewed and approved by Ethics Committee of the First Affiliated Hospital of Jinan University. Written informed consent to participate in this study was provided by the participants’ legal guardian/next of kin.

## Author Contributions

YQL and XH contributed to the concept development and study design. LX, LL, DLY, XBZ, YKZ, and SHS performed the laboratory studies. JL, ZYJ, XFZ, RHZ, YHL, MML, and XH collected the clinical samples. LL collected the clinical information of patients. LX and YQL drafted the manuscript. SHC managed the laboratory reagents and financial affairs. LX and YQL helped modify the manuscript. All authors contributed to the article and approved the submitted version.

## Funding

This study was supported by grants from the National Natural Science Foundation of China (Nos. 82000108, 82070152, 91642111, 81890991, and 81800143), China postdoctoral science foundation (2018M640884), Guangdong Provincial Outstanding Young Medical Talents Supporting Research Foundation (KJ012019459), Guangdong Provincial Natural Science Foundation (2020A1515110310), Guangzhou Science and Technology Project (201807010004 and 201803040017), and Key Laboratory for Regenerative Medicine of Ministry of Education Project (ZSYXM202001).

## Conflict of Interest

Author YKZ was employed by company Tianhe Nuoya Bio-Engineerinig Co. Ltd.

The remaining authors declare that the research was conducted in the absence of any commercial or financial relationships that could be construed as a potential conflict of interest.

## Publisher’s Note

All claims expressed in this article are solely those of the authors and do not necessarily represent those of their affiliated organizations, or those of the publisher, the editors and the reviewers. Any product that may be evaluated in this article, or claim that may be made by its manufacturer, is not guaranteed or endorsed by the publisher.

## Supplementary Material

Supplementary Figure 1 Expression frequency of TIGIT on the PB CD4^+^ and CD8^+^ subsets of the HI, DN-AML, and CR-AML cohorts in the young and old age groups. Flow-cytometry analysis shows that the expression frequency of TIGIT increased in the PB CD4^+^ population of DNy patients when compared with HIy.

Supplementary Figure 2 The absolute number of CD3^+^, CD4^+^, and CD8^+^ T cells in the PB and BM of the HI and AML cohorts. The total CD3^+^ T cell number in PB heavily decreased in CR and R/R patients who received induction chemotherapy, and total CD3^+^ T cells in the BM increased in DN-AML patients when compared with HIs.
