# Supplementary figures and images for: PD-1 and TIGIT Are Highly Co-Expressed on CD8+ T Cells in AML Patient Bone Marrow
Source: Front Oncol. 2021 Aug 18;11:686156. doi: 10.3389/fonc.2021.686156 (PMC8416522; doi:10.3389/fonc.2021.686156)

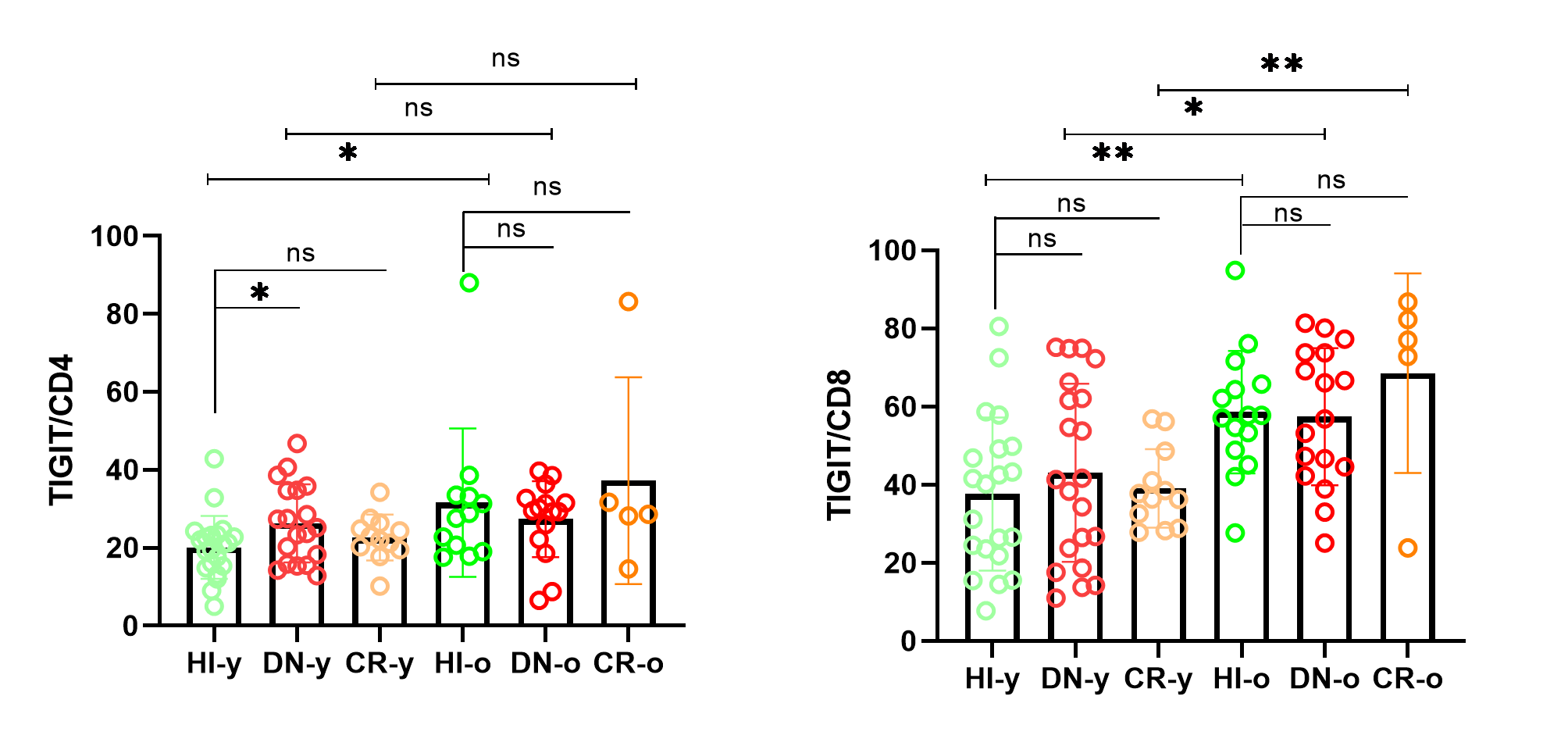

Supplement: Supplementary Figure 1 — Expression frequency of TIGIT on the PB CD4+ and CD8+ subsets of the HI, DN-AML, and CR-AML cohorts in the young and old age groups. Flow-cytometry analysis shows that the expression frequency of TIGIT increased in the PB CD4+ population of DNy patients when compared with HIy. [file Image_1.tif]

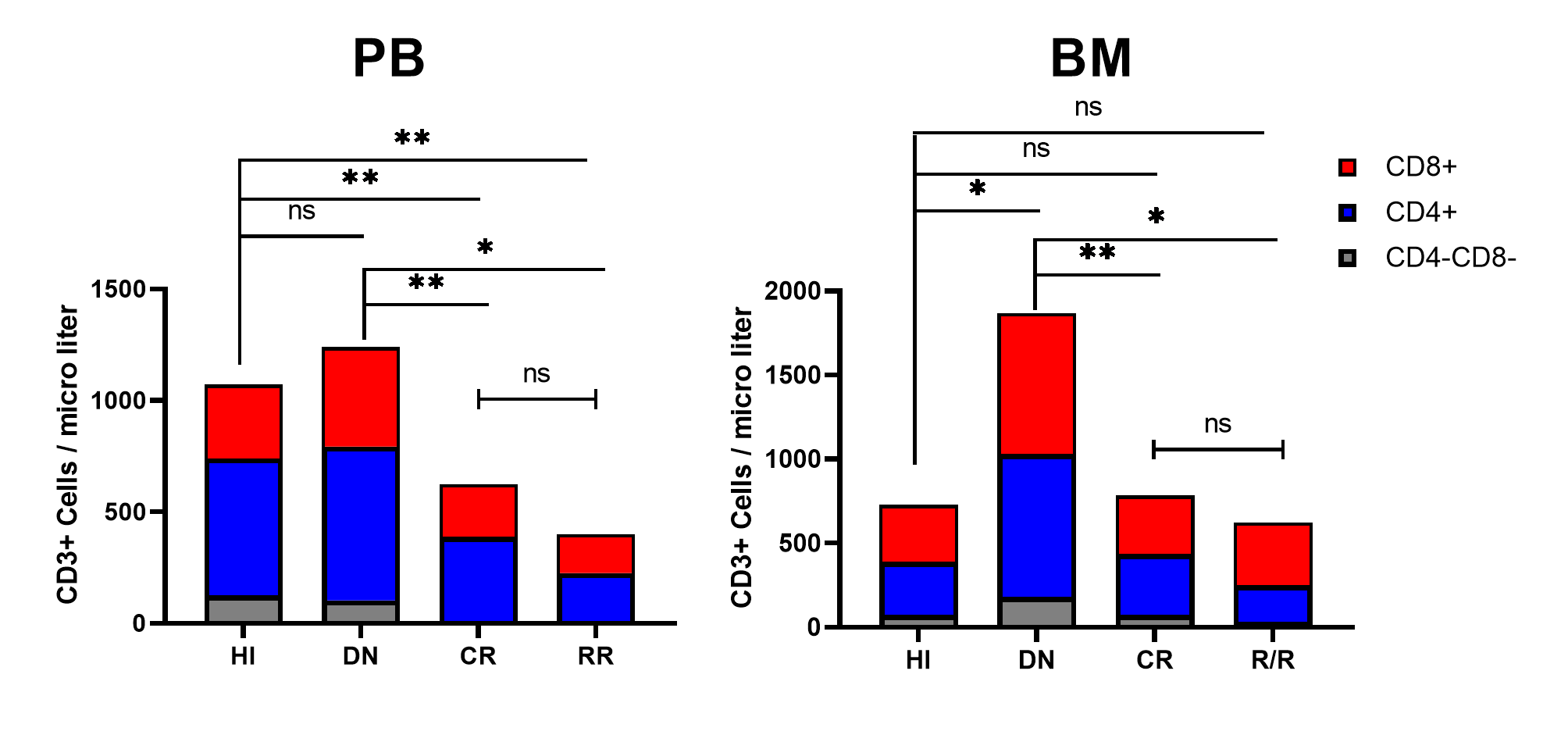

Supplement: Supplementary Figure 2 — The absolute number of CD3+, CD4+, and CD8+ T cells in the PB and BM of the HI and AML cohorts. The total CD3+ T cell number in PB heavily decreased in CR and R/R patients who received induction chemotherapy, and total CD3+ T cells in the BM increased in DN-AML patients when compared with HIs. [file Image_2.tif]
